# Household disinfection practices by women living in Egypt during the 2020 COVID-19 lockdown and the association of information sources and suspected bleach toxicity

**DOI:** 10.1186/s12889-022-14570-2

**Published:** 2022-11-19

**Authors:** Maha Farid, Rania Talaat, Valerie Pacino, Hyo Jung Tak, Wael ElRayes

**Affiliations:** 1 Department of Forensic Medicine and Clinical Toxicology, Faculty of Medicine, Helwan University, Cairo, Egypt; 2 Department of Medical Microbiology and Immunology, Faculty of Medicine, Helwan University, Cairo, Egypt; 3 Department of Health Services Research and Administration, College of Public Health, University of Nebraska Medical Center, Omaha, NE USA

**Keywords:** Egypt, COVID-19, Social media, Toxicity, Bleach

## Abstract

**Introduction:**

The spread of contradictory health information was a hallmark of the early COVID-19 pandemic. Because of a limited understanding of the disease, its mode of transmission, and its pathogenicity, the public turned to easily accessible and familiar sources of information. Some of these sources included wrong or incomplete information that could increase health risks and incidents of toxicity due to improper information about the usage of disinfectants. The objective of this study was to assess the relationship between sources of information about the COVID-19 pandemic, the related household cleaning and disinfection practices among adult women living in Egypt, and the associated adverse effects of bleach toxicity during a national lockdown.

**Methods:**

Through a self-administered online survey, 452 adult women (18 years and older) living in Egypt were recruited from 13 cities between 4 June 2022 and 4 July 2022 to answer the questionnaire. The questionnaire included (41) questions in Arabic and collected data about respondents' household cleaning and disinfection practices to prevent the spread of the SARS-CoV-2 virus and protect their families during the lockdown that started in Egypt in March 2020.

**Results:**

The study found that 88.1% (*n *= 398) of participants reported increased use of disinfectants during the lockdown. Women who chose social media as their primary source of information to learn about disinfection practices reported an increased frequency of respiratory symptoms associated with bleach toxicity (correlation coefficient = 0.10, *p*-value = 0.03), followed by women who depended on relatives and friends as the primary source of information (correlation coefficient = 0.10, *p*-value = 0.02).

**Conclusion:**

This study showed that social media is an easily accessible, efficient and fast communication tool that can act as a primary source for individuals seeking medical information compared to other media platforms (e.g., websites, T.V., satellite channels). However, better regulations and monitoring of its content may help limit the harms caused by the misinformation and disinformation spread by these popular platforms, particularly in times of uncertainty and upheaval.

**Supplementary Information:**

The online version contains supplementary material available at 10.1186/s12889-022-14570-2.

## Introduction

The global COVID-19 pandemic exposed the world to unprecedented realities. From contradictory medical information about the virus, to controversial facts about its mode of transmission, to failures of governments to control the spread of the disease, to an unprecedented scramble on social media to seek information about the disease, the world faced serious threats to its confidence in its governments and healthcare systems, to national security, and to social stability [1, 2]. To control the spread of the diseases, many countries imposed a series of actions that escalated from partial lockdowns to complete curfews, to bans on public gatherings, to delayed academic activities in schools and colleges, and border closures [1]. Similarly, the Egyptian government issued and enacted 44 laws and decrees in response to the pandemic that included various national restrictions that culminated with a nationwide lockdown.

Many felt overwhelmed with the conflicting information and struggled to assess the credibility and reliability of different health information sources [2, 3]. While some sources were effective in communicating timely and accurate information to the public, other sources widely distributed misinformation and disinformation that may have adversely harmed the public [2–4]. At the same time, people were advised to stay home and refrain from seeking in-person medical care to avoid being infected by the coronavirus, SARC-CoV-2. Many people who once sought trusted sources for health information turned to social media platforms and other online sources for dubious health and medical advice [2].

The frenzy of information about how to prevent the spread of SARC-CoV-2 led people to purchase and use disinfectants and personal protective equipment (PPE) that they had no experience or training to use [5]. One alarming trend was captured by the *Morbidity and Mortality Weekly Report* (MMWR), which reported a sharp increase in the number of calls to poison control centers in the United States, seeking advice and medical management for toxicity incidents related to using disinfectants and household cleaners since the onset of the COVID-19 pandemic [6]. Another study showed a significant increase in calls to poison control centers in Canada regarding the use of cleaning products and disinfectants during the first three months of 2020, estimating a 400% increase in comparison to the same period in the previous year. Of these calls, 42% and 12% were related to household use of bleach and chlorine gas, respectively [7]. However, limited data is available about the adverse effects related to household cleaning and disinfection practices in lower-resource countries around the world.

This study aims to investigate the changes in household disinfection practices by women living in Egypt during the 2020 COVID-19 lockdown and the associated increase of adverse effects from toxicity by bleach and similar products. Our assumption is this was partially caused by panic during the lockdown and the spread of misinformation and disinformation on social media platforms about household cleaning and disinfection practices to prevent the transmission of the SARS-CoV-2 virus. Results from this study enforce the critical role that social media plays in our modern life and should call for improved government oversight and control of social platforms, especially during public health emergencies. This also calls for social media companies to better self-governance and content monitoring to limit as much as possible the negative impact of mis and disinformation. To our knowledge, this is the first study investigating such relationships in Egypt.

## Materials and methods

All adult women (18 years old and older) living in Egypt during the time of the COVID-19 lockdown that began in March 2020 were eligible for inclusion. The questionnaire was open for a month from June 4 through July 4, 2020.

No personal identification data was collected (names or contact information), participation was voluntarily, and informed consent was obtained from all subjects through a consent statement at the beginning of the online questionnaire form. This study protocol was reviewed by the Research Ethics Committee for Human and Animal Research at the Faculty of Medicine at Helwan University and was approved (serial no. 31-2022).

The authors used Google Forms to develop an English questionnaire of 41 multiple-choice questions. The survey was developed de novo, based on the research questions and the aims of this study. For all survey questions, respondents selected from categorical options. The survey was reviewed and verified by experts in the field of forensic medicine and clinical toxicology. The survey tool was then translated into Arabic and tested to ensure consistency and reliability with the English version. The final Arabic questionnaire was sent to the target population via the social media platforms Facebook and WhatsApp. We used convenience and snowball sampling approaches to send the survey to adult women living in Egypt with no restrictions on which governorate the women lived in. The initial convenience sample included family and friends through WhatsApp groups. The snowballing approach was done through the initial convenience sample and through posting the survey in several Facebook groups for Arabic speaking women that are known to include members from different socioeconomic and educational levels. The number of members of these groups ranges between 19,000 and 39,000. Completed surveys were collected via the Google Form online self-administered questionnaire.

Survey questions included sources of information used to learn about the COVID-19 pandemic, and the household cleaning and disinfection practices respondents undertook to prevent the transmission of the SARS-CoV-2 virus. Additionally, the survey assessed the frequency of household cleaning and disinfection practices and the usage of different products compared to the months preceding the pandemic, the occurrence of adverse effects related to bleach toxicity during the lockdown, and in the case of suspected toxicity, how women reached out for medical care for their family members. Additionally, some demographic data about the participants such as age group, location in Egypt, number of family members in each household, and the education level was collected.

The exposure variable was primary information sources, and the outcome variables were development of symptom, type of symptom, and use of PPE. For data analysis, summary statistics demonstrate the distribution of each outcome. We did not perform any univariate statistical analysis, as we had no hypothesized proportions of the category of each variable when we collected survey responses. We constructed a binary variable for each category of primary sources for obtaining information to use disinfectants (e.g., CDC or WHO website) and development of symptoms of bleach toxicity (e.g., any symptom) and performed Pearson’s Phi test for each pair of binary variables [8, 9]. All analysis was conducted using Stata v.16 MP.

## Results

A total of 454 respondents completed the questionnaire. Two respondents were excluded from the analysis, because they resided outside Egypt during the lockdown that started March 2020.

Our data shows that 76.9% (*n* = 351) of the respondents were in the age group 26–45 followed by the age group 46–59 at 14.5% (*n* = 66). Respondents were from 14 urban and rural governorates out of 26 governorates in Egypt, including Cairo, Giza, Alexandria, the Delta, and Upper Egypt. Cairo residents represented 67.3% (*n* = 304) of the total respondents, while 8.9% (*n* = 40) were from Giza Governorate. Of all participants, 67.7% (*n* = 306) have a college degree, 14.6% (*n* = 66) have a master’s level education, and 13.3% (*n* = 60) have a doctoral level education. Survey results show that 29% (*n* = 131) of participants work in the healthcare field in different capacities.

Of all the survey respondents, 73.8% (*n* = 334) reported an increased frequency of household cleaning during the COVID-19 lockdown, of whom 88.1% (*n* = 298) increased their use of disinfectants. Furthermore, about 79.4% (*n* = 358) used bleach and 14.2% (*n* = 58) of them mixed bleach with other products rather than water.

**Fig. 1:**
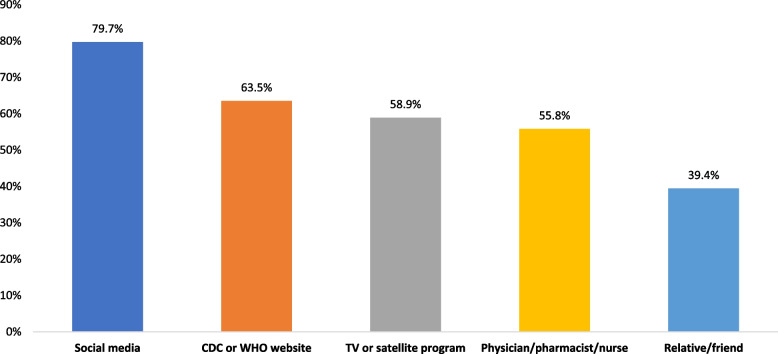
Primary source(s) for obtaining information to use disinfectants during the pandemic lockdown. Includes multiple sources of information that are not mutually exclusive (number of respondents: social media: 360, CDC or WHO websites: 287, TV or Satellite program: 266, Physician/pharmacist/nurse: 252, Relative/friend: 178)

The majority of participants, 91% (*n* = 411) obtained information about how to use disinfectants from multiple sources. The most common sources of information were social media (79.7%, *n* = 360) followed by CDC or WHO websites (63.5%, *n* = 287), and TV/satellite (58.9%, *n* = 266) (see Fig. 1). When we asked about why respondents chose this source as the primary source of information, 46% (*n* = 208) of the respondents answered that the main reason was the ease of getting information from that source, followed by 44% (*n *= 199) whose main reason was the trust in that source of information. Although respondents used different sources to get information about the use of disinfectants, the CDC or WHO websites were reported to be the most trusted source (62%, *n* = 280) whereas 56% (*n* = 253) of those who consulted with a physician, pharmacist or nurse trusted the information. 52% (*n* = 253) of the respondents who used social media (Facebook, WhatsApp) trusted these sources versus 48% (*n* = 217) who used Arabic TV and satellite Programs. Consulting a relative or a friend showed the lowest level of trust.

**Fig. 2:**
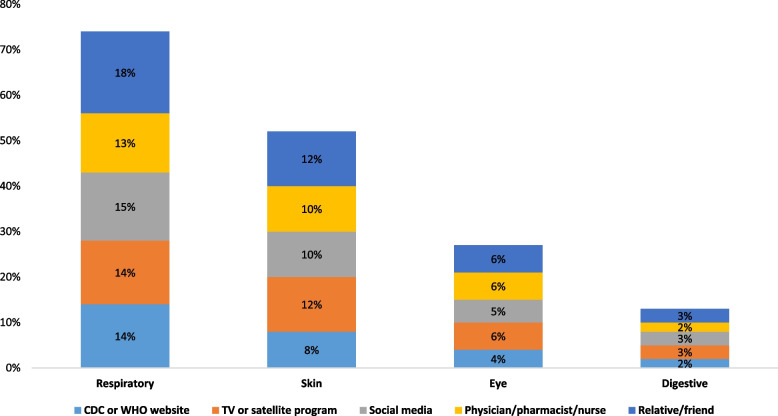
Sources of information and development of adverse medical symptoms, by type. * Symptoms were not mutually exclusive in response options

**Fig. 3:**
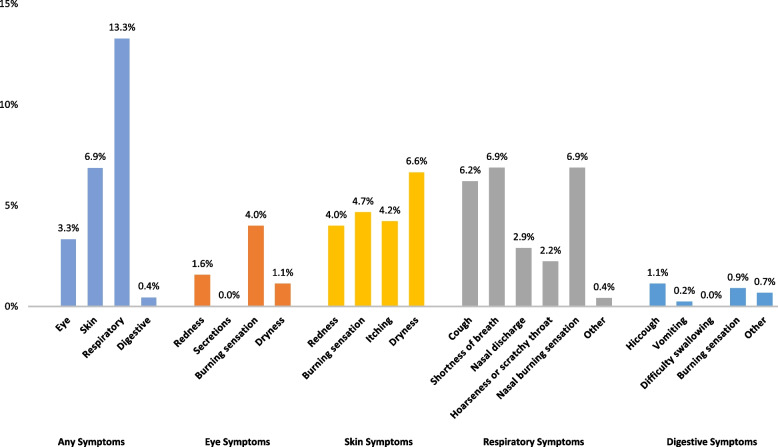
Primary symptoms, overall and by symptom type

Among the type of symptoms, respiratory symptoms were the most frequently reported, followed by skin, eye, and digestive symptoms. Some individuals reported multiple symptoms. The order of frequency of symptoms was consistent across sources of information (Fig. 2). The primary symptom type for each system was shortage of breath (6.9%) and nasal burning sensation (6.9%) for respiratory, dryness for skin (6.6%), burning sensation for eyes (4.0%), and hiccup for digestive (1.1%) (Fig. 3).

We found that more individuals developed respiratory symptoms of bleach toxicity when social media was their main source of information to learn about cleaning and disinfection practices (correlation coefficient = 0.10, *p*-value = 0.03) followed by a relative or a friend (correlation coefficient = 0.10, *p*-value = 0.02). In addition, individuals were more likely to develop any symptom (i.e., eye, skin, respiratory, or digest) when they obtained information from a relative and a friend (correlation coefficient = 0.10, *p*-value = 0.04). Obtaining information from more reputable public-facing health sources, such as CDC or WHO website, TV and satellite, or a health professional were not associated with development of symptoms (Table 1).

**Table 1:** Correlation between primary source(s) for obtaining information to use disinfectants and development of symptoms of bleach toxicity

	Any symptom	Eye symptoms	Skin symptoms	Respiratory symptoms	Digestive symptoms
*Primary Source(s) of Information*	*(n = 452)*	*(n = 452)*	*(n = 452)*	*(n = 452)*	*(n = 452)*
CDC or WHO website					
Correlation coefficient	0.0024	-0.0545	-0.0517	0.0036	-0.0294
*p*-value	0.959	0.248	0.272	0.939	0.534
TV or satellite program					
Correlation coefficient	0.0549	0.0709	0.0873	0.0277	0.0154
*p*-value	0.244	0.132	0.064	0.558	0.745
Social media					
Correlation coefficient	0.0771	0.017	0.0515	**0.1032**	0.0442
*p*-value	0.102	0.718	0.274	0.028*	0.349
Physician, pharmacist, nurses					
Correlation coefficient	-0.0058	0.0239	0.0156	-0.0132	-0.0038
*p*-value	0.902	0.613	0.741	0.780	0.935
Relative and friend					
Correlation coefficient	**0.0985**	0.0194	0.0782	**0.1057**	0.049
*p*-value	0.036*	0.681	0.097	0.025*	0.298

* *p*-value < 0.05 is considered significant and is noted above

**Fig. 4:**
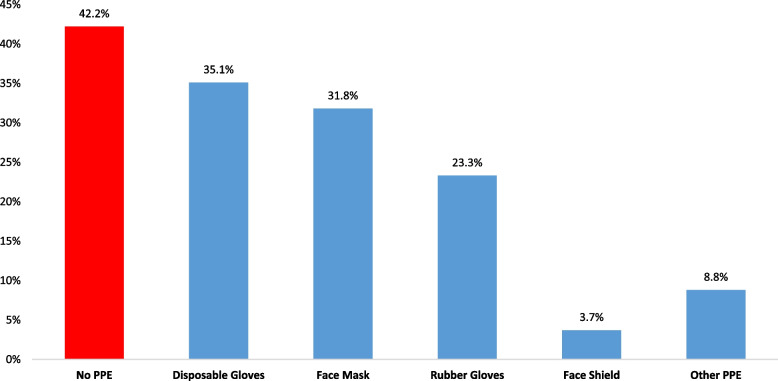
Personal protective equipment used to reduce bleach toxicity, by PPE type

Among respondents who increased the frequency of the use of bleach in household cleaning (*n* = 365), 42.2% did not use any PPE. The most frequently used PPE were disposable gloves (35.1%), face mask (31.8%), and rubber/latex gloves (23.3%) (Fig. 4).

When we examined the correlation between source of information and other variables (e.g., the use of personal protective equipment while disinfecting the household, whether the respondent or any other family member in the same household experienced any adverse effects to bleach, the age of the respondent, the level of education, and the occupation of the person completing the survey), we found several significant correlations. Although most respondents used social media as their primary source of information, individuals with higher education levels and individuals with medical background were less likely to trust information from social media (*p*-value 0.07, 0.001 respectively). Although 40% of respondents used friends and relatives as a source of information, older people, people with higher education levels, and individuals with medical background were less likely to trust their information (*p*-value 0.06, < 0.0001, 0.0001 respectively).

## Discussion

In this study, we assessed household cleaning and disinfection practices and suspected bleach toxicity among adult women and their household members living in Egypt. Across different socioeconomic levels, women remain responsible for household cleaning in Egypt. Data from poison control centers in Egypt are not publicly available to include in this study. The survey was distributed in Arabic to be easily understood by our target group across different educational strata.

To our knowledge, this is the only study that investigated the sources of information used by women living in Egypt during the COVID-19 pandemic and analyzed the demographics as confounding factors to the data. Our findings show that older women, individuals with higher education levels, and individuals with medical background do not trust the information drawn from social media sources, though they still referenced these sources. Additionally, most of the respondents were neither old age nor with medical background, which is reflective of the demographic distribution in Egypt. This shows that a number of social media users are exposed and vulnerable to the erroneous information provided through these sources. In fact, according to a news report, one of the most popular social media platforms started posting warnings about medical information concerning COVID-19 due to the reported misinformation during and after the pandemic, and directing users to other trustable websites with information maintained and monitored by international health organizations such as the WHO website [10].

Our findings are consistent with those of other studies performed in North America where respondents reported increased usage of bleach for household cleaning and disinfection practices [6, 7]. Women who reported that they used social media as their main source of information during the lockdown showed statistically significant increase in respiratory symptoms as the primary manifestation of bleach toxicity. Similarly, Chary and colleagues found geospatial correlation between misinformation on social media and calls to poison centers inquiring about toxicity with household cleaners in the Greater Boston Area [11].

Kouzy et al. reported in early 2020 that disseminating medical misinformation related to the global pandemic on social media was alarming and suggested early interventions to prevent further complication of the disease [12]. Even before the start of the COVID-19 pandemic, studies analyzing medical information on social media (e.g., Twitter) found that approximately half of this information is not evidence-based and classified as false by experts [13].

Bleach is an inexpensive, readily available product that people often integrate into household cleaning and disinfection practices. Because of its ubiquity, most people perceive it is a relatively safe product and tend to be less cautious when using it. Our data showed that only two-thirds of respondents (67.8%) used PPE while cleaning and disinfecting their homes, the majority of those (35.1%) used only plastic disposable gloves (they did not use face masks) which could partially explain why respiratory symptoms was the highest among all other symptoms reported in this study. Our data is consistent with the findings by Dindarloo et al., about household cleaning and disinfection practices in Iran. The authors reported that 55.3% of Iranian respondents depended on social media as their main source of information to learn about disinfection practices during the lockdown. However, in their study, skin itching was the most common symptom associated with suspected toxicity [14].

Our findings are consistent with alarming findings in previous similar studies. People tend to rely on social media as a common source for medical information, because these channels of communication are familiar and readily accessible. Given the use of mobile phones and other communication devices (i.e., tablets), social media is now a presence in many people’s lives. With the lack of laws or systems in place to control what is published on these sources, individuals will continue to have access to incorrect or false medical information, which could lead to serious medical complications. Social media companies should work collaboratively with national and transnational governments to establish parameters, build regulations, implement mechanisms, and enforce laws that control the medical information disseminated through these sources.

Interestingly enough, the most trusted sources of information were the CDC or WHO websites, however, and probably because they were either hard to reach or individuals did not know about them, more initiatives and efforts should be directed to increase the availability of these trusted sites on social media, especially in fast-moving crises like this pandemic. More funding should be allocated to education and awareness campaigns to inform individuals about these trusted and reliable sources.

The results of this study are consistent with other findings that the increased use of disinfectants during the COVID-19 pandemic was associated with increased rates of suspected toxicity incidents. People obtained their information from a variety of sources but a common one was social media. The dissemination of medical information on social media is a double-edged sword and regulatory actions should be considered to guard against the misuse of these powerful and far-reaching tools.

## Limitations

This work had several limitations that were primarily driven by the timing of conducting the survey during the complete lockdown in Egypt in 2020. These limitations included relying on self-identification of symptoms by the respondents and not relying on clinical or documented diagnosis. Another limitation is related to the small sample size, however this was driven by the fact that the survey was conducted during the complete lockdown time in Egypt, nevertheless, the sample size is comparable to similar studies conducted by other researchers in other countries during the same period. Additionally, we relied solely on social media platforms for distributing the survey, so people who does not use social media were not included [6].

## Supplementary Information

**Additional file 1:** **Supp. Fig 1.** The disinfectants used by participants since the beginning of the lockdown. **Supp. Fig 2.** The responses to the question: if you used bleach for disinfection during the lockdown, did you mix with any other substances?. **Supp. Fig 3.** The responses to the question: How did you use the cleaning products and disinfectants in your household during the lockdown?. **Supp. Fig 4.** The responses to the question: Did you use any personal protective equipment for these chemical products while disinfecting your household during the lockdown for Corona virus?. **Supp. Table 1.** The answer to the question: What is the socioeconomic status (i.e., highest level of education) of the person who filled in the survey?.  **Supp. Table 2.** The answer to the question: What is the age group of the person who filled in the survey?. **Supp. Table 3.** The answer to the question: What is the geographic location of the person who filled in the survey?. **Supplement Data for Figure 2.** Information of multiple responses. **Supplement Data for Figure 3.** Information of multiple responses. **Supplement for Figure 4.** Information of multiple responses. The Survey Question in English.

## Data Availability

The datasets used and/or analyzed during the current study available from the corresponding author on reasonable request.
