# Supplementary material for: Household disinfection practices by women living in Egypt during the 2020 COVID-19 lockdown and the association of information sources and suspected bleach toxicity
Source: BMC Public Health. 2022 Nov 19;22:2125. doi: 10.1186/s12889-022-14570-2 (PMC9675102; doi:10.1186/s12889-022-14570-2)
Supplement: Supplementary file 1 — Additional file 1: Supp. Fig 1. The disinfectants used by participants since the beginning of the lockdown. Supp. Fig 2. The responses to the question: if you used bleach for disinfection during the lockdown, did you mix with any other substances?. Supp. Fig 3. The responses to the question: How did you use the cleaning products and disinfectants in your household during the lockdown?. Supp. Fig 4. The responses to the question: Did you use any personal protective equipment for these chemical products while disinfecting your household during the lockdown for Corona virus?. Supp. Table 1. The answer to the question: What is the socioeconomic status (i.e., highest level of education) of the person who filled in the survey?. Supp. Table 2. The answer to the question: What is the age group of the person who filled in the survey?. Supp. Table 3. The answer to the question: What is the geographic location of the person who filled in the survey?. Supplement Data for Figure 2. Information of multiple responses. Supplement Data for Figure 3. Information of multiple responses. Supplement for Figure 4. Information of multiple responses. The Survey Question in English. [file 12889_2022_14570_MOESM1_ESM.pdf]

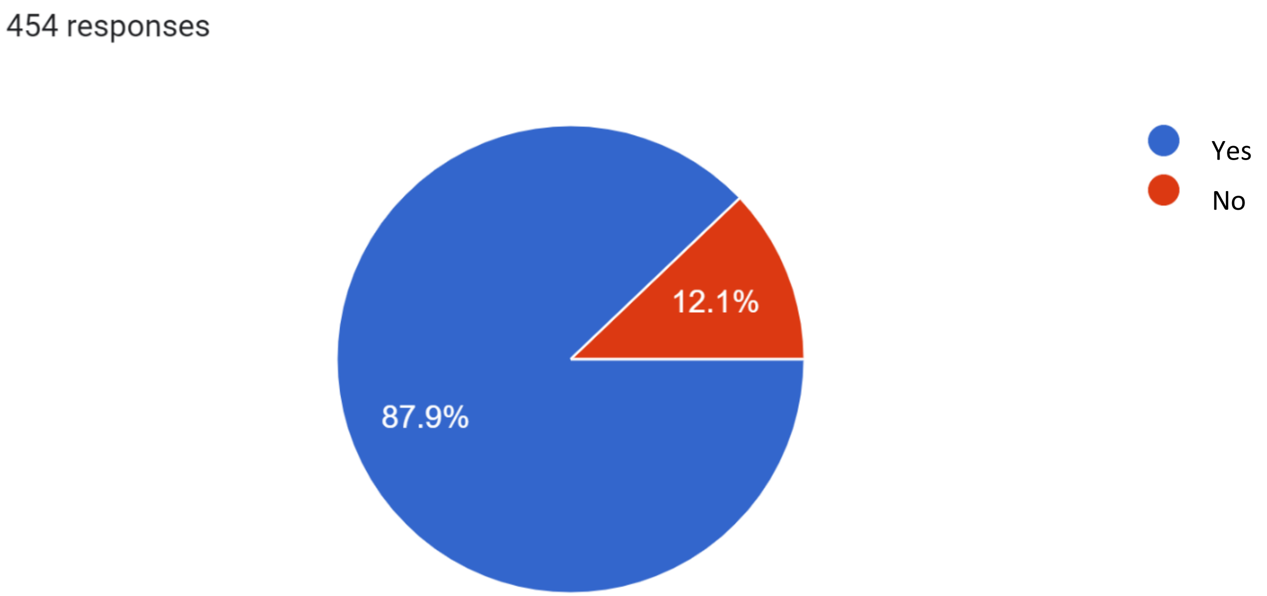

Supp. Fig 1: The disinfectants used by participants since the beginning of the lockdown.

(Soap: 262 (65.7 %), Dettol: 120 (30.1 %), Lysol: 8 (2 %), Bleach: 360 (90.2 %), Alcohol: 298 (74.7 %), Others: 22 (5.5 %)).

N.B. results are not mutually exclusive.

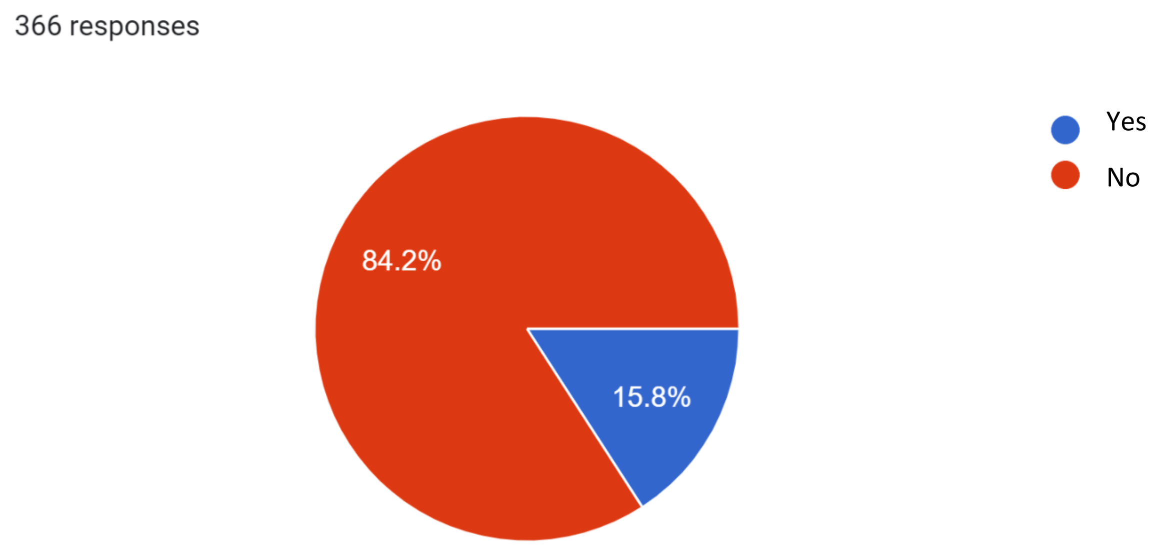

Supp. Fig 2: The responses to the question: if you used bleach for disinfection during the lockdown, did you mix with any other substances? Answers: No 308 (84.2%), Yes 58 (15.8%).

The number of the responses (366) is less than the total number of resposes of the survey (454) as this question appears only to participants responded with “yes” to a previous question asking about increased use of disinfectants since the beginning of the lock down (see questionnair questions attached).

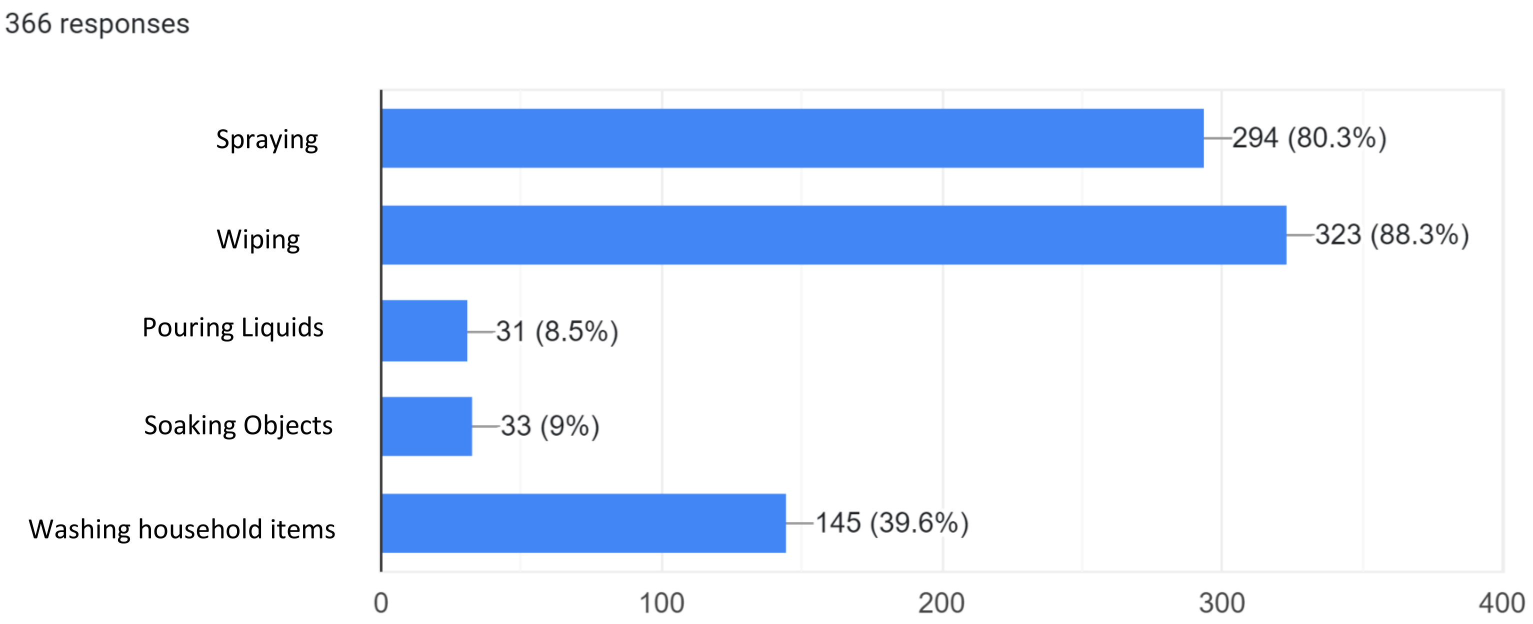

Supp. Fig 3: The responses to the question: How did you use the cleaning products and disinfectants in your household during the lockdown? You can choose all what applies for you? Answers: Spraying 294 (80.3%), Wiping 323 (88.3%), Pouring liquids 31 (8.5%), Soaking objects 33 (9%), Washing household objects 145 (36.6%).

N.B. results are not mutually exclusive. The number of the responses (366) is less than the total number of resposes of the survey (454) as this question appears only to participants responded with "yes" to a previous question asking about increased use of disinfectants since the beginning of the lock down (see questionnair questions attached).

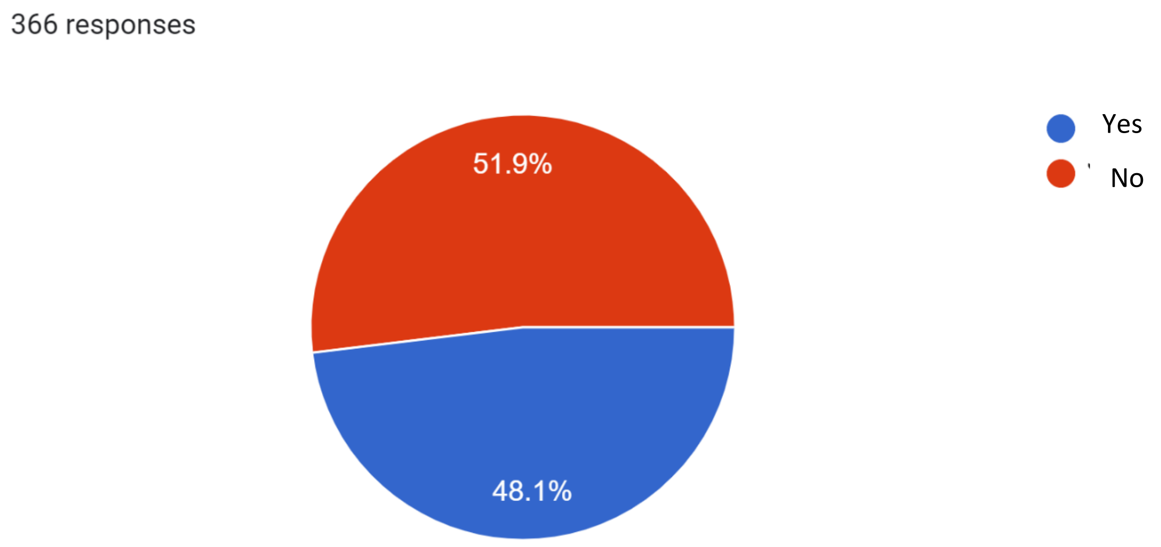

Supp. Fig 4: The responses to the question: Did you use any personal protective equipment for these chemical products while disinfecting your household during the lockdown for Corona virus? Answers: No 190 (51.9%), Yes 176 (48.1%).

The number of the responses (366) is less than the total number of resposes of the survey (454) as this question appears only to participants responded with “yes” to a previous question asking about increased use of disinfectants since the beginning of the lock down (see questionnair questions attached).

	Highest degree	
Category	No.	(%)
1: Secondary Degree (High school) or less	1	(4.4)
2: University (College) Degree	2	(67.7)
3: Master's (Level)Degree	3	(14.6)
4: Doctoral (Level) Degree	4	(13.3)
Total	452	(100.0)

Supp. Table 1 : The answer to the question: What is the socioeconomic status (i.e., highest level of education) of the person who filled in the survey?

Age Group	No	%
18-25 yrs	24	(5.3)
26-45 yrs	351	(77.3)
46-59 yrs	66	(14.5)
60 or more yrs	11	(2.4)
Total	452	(100.0)

Supp. Table 2 : The answer to the question: What is the age group of the person who filled in the survey?

	Geographic location	
	No.	(%)
0: ***	23	(5.1)
1: Cairo	304	(67.3)
2: Giza	40	(8.9)
3: Alexandria	3	(0.7)
4: Al-Qalyubia	7	(1.6)
5: Al-Monufia	7	(1.6)
6: Al-Daqahlia	9	(2.0)
8: Al-Sharqia	6	(1.3)
10: Damiatta	4	(0.9)
11: Kafr Al-Sheikh	2	(0.4)
18: Al-Menia	2	(0.4)
19: Assiut	32	(7.1)
20: Sohag	8	(1.8)
21: Qena	1	(0.2)
22: Luxor	1	(0.2)
23: Aswan	2	(0.4)
missing	1	(0.2)
Total	452	(100.0)

Supp. Table 3 : The answer to the question: What is the geographic location of the person who filled in the survey?

N.B. This question was the only free answer question in the questionnaire, therefore, the category 0*** represents the answers that were not clear and did not fit into the categories listed in the table.

Supplement Data for Figure 2. Information of multiple responses

Any symptom	Freq.	Percent	Eye	Freq.	Skin	Freq.	Respiratory	Freq.	Digestive	Freq.
**0**	5	1.1	1, 2	1	1, 2	1	0	1	0	1
**1**	12	2.7	1, 2, 3	4	1, 2, 3	4	1, 2, 3	11	1, 2, 3	2
**1, 2**	8	1.8	1, 2, 3, 4	1	1, 2, 3, 4	3	1, 2, 3, 4	3	1, 2, 3, 4, 5	2
**1, 2, 3**	46	10.2	1, 2, 3, 4, 5	3	1, 2, 3, 4, 5	5	1, 2, 3, 4, 5	7	1, 2, 3, 5	1
**1, 2, 3, 4**	35	7.7	1, 2, 4	1	1, 2, 3, 5	4	1, 2, 3, 5	5	1, 3, 4	1
**1, 2, 3, 4, 5**	37	8.2	1, 3, 4	1	1, 2, 4	2	1, 2, 4	1	2, 3, 4, 5	2
**1, 2, 3, 5**	20	4.4	1, 4	1	1, 3, 4	2	1, 3	1	3	1
**1, 2, 4**	17	3.8	2	1	1, 3, 5	1	1, 3, 4	2	3, 4, 5	1
**1, 2, 4, 5**	3	0.7	2, 3, 4	1	1, 4	2	1, 3, 4, 5	4	Total	11
**1, 2, 5**	3	0.7	2, 3, 4, 5	3	2	1	1, 3, 5	1		
**1, 3**	18	4.0	2, 3, 5	2	2, 3	1	1, 4	3	Note:	
**1, 3, 4**	33	7.3	3	1	2, 3, 4	3	1, 5	1	1 = CDC or WHO website	
**1, 3, 4, 5**	21	4.7	3, 4	1	2, 3, 4, 5	4	2, 3, 4	3	2 = TV or satellite program	
**1, 3, 5**	9	2.0	3, 4, 5	2	2, 3, 5	3	2, 3, 4, 5	4	3 = Social media	
**1, 4**	18	4.0	Total	23	3	2	2, 3, 5	4	4 = Physician/pharmacist	
**1, 4, 5**	5	1.1			3, 4, 5	4	3	3	5 = Relative/friend	
**1, 5**	2	0.4	Note:		3, 5	1	3, 4	1		
**2**	5	1.1	1 = CDC or WHO website		Total	43	3, 4, 5	5		
**2, 3**	31	6.9	2 = TV or satellite program				3, 5	1		
**2, 3, 4**	15	3.3	3 = Social media		Note:		Total	61		
**2, 3, 4, 5**	27	6.0	4 = Physician/pharmacist		1 = CDC or WHO website					

**2, 3, 5**	15	3.3		5 = Relative/friend			2 = TV or satellite program			Note:				
**2, 4**	1	0.2					3 = Social media			1 = CDC or WHO website				
**2, 4, 5**	2	0.4					4 = Physician/pharmacist			2 = TV or satellite program				
**2, 5**	1	0.2					5 = Relative/friend			3 = Social media				
**3**	14	3.1								4 = Physician/pharmacist				
**3, 4**	10	2.2								5 = Relative/friend				
**3, 4, 5**	18	4.0												
**3, 5**	11	2.4												
**4**	6	1.3												
**4, 5**	4	0.9												
**Total**	452	100.0												
**Note:**														
**1 = CDC or WHO website**														
**2 = TV or satellite program**														
**3 = Social media**														
**4 = Physician/pharmacist**														
**5 = Relative/friend**														

**Supplement Data for Figure 3. Information of multiple responses**																			
**Any symptom**	Freq.	Percent		Eye symptoms	Freq.	Percent		Skin symptoms	Freq.	Percent		Respiratory symptom	Freq.	Percent		Digestive symptom	Freq.	Percent	
**No symptom**	383	84.73		No symptom	429	94.91		No symptom	409	90.49		No symptom	391	84.73		No symptom	441	84.73	
**1, 2**	1	0.22		1	4	0.88		1	2	0.44		1	8	1.77		1	3	0.66	
**1, 2, 3**	7	1.55		1, 3	1	0.22		1, 2, 3	1	0.22		1, 2	4	0.88		1, 2	1	0.22	
**1, 3**	6	1.33		1, 3, 4	2	0.44		1, 2, 3, 4	5	1.11		1, 2, 3	2	0.44		1, 4	1	0.22	
**1, 3, 4**	1	0.22		3	13	2.88		1, 2, 3, 4	1	0.22		1, 2, 3, 4, 5	1	0.22		4	3	0.66	
**2**	8	1.77		3, 4	2	0.44		1, 2, 4	2	0.44		1, 2, 3, 5	1	0.22		5	3	0.66	
**2, 3**	14	3.1		4	1	0.22		1, 3	1	0.22		1, 2, 4, 5	2	0.44		Total	452	100	
**2, 3, 4**	1	0.22		Total	452	100		1, 3, 4	1	0.22		1, 3	1	0.22					
**3**	31	6.86						1, 4	5	1.11		1, 3, 4	2	0.44		Note:			
**Total**	452	100		Note:				2	5	1.11		1, 3, 4	1	0.22		1 = Hicough			
				1 = Redness				2, 3	2	0.44		1, 4	2	0.44		2 = Vomiting			
**Note:**				2 = Secretions				2, 3, 4	2	0.44		1, 4, 5	1	0.22		3 = Difficulty swallowing			
**1 = Eye**				3 = Burning sensation				2, 4	3	0.66		1, 5	3	0.66		4 = Burning sensation			

**2 = Skin**				4 = Dryness				3	2	0.44		2	10	2.21		5 = Other			
**3 = Respiratory**								3, 4	4	0.88		2, 3, 4, 5	1	0.22					
**4 = Digestive**								4	7	1.55		2, 3, 5	3	0.66					
								Total	452	100		2, 5	6	1.33					
												2, 5, 6	1	0.22					
								Note:				3, 5	1	0.22					
								1 = Redness				5	10	2.21					
								2 = Burning sensation				5, 6	1	0.22					
								3 = Itching				Total	452	100					
								4 = Dryness											
												Note:							
												1 = Cough							
												2 = Shortness of breath							
												3 = Nasal discharge							
												4 = Hoarseness or scratch throat							
												5 = Nasal burning sensation							
												6 = Other							

Supplement for Figure 4. Information of multiple responses

Personal protective equipment	Freq.	Percent	Cum.
0	147	40.27	40.27
1	21	5.75	46.03
1, 2	20	5.48	51.51
1, 2, 3	7	1.92	53.42
1, 2, 3, 4	1	0.27	53.7
1, 2, 3, 5	2	0.55	54.25
1, 2, 4	5	1.37	55.62
1, 2, 5	2	0.55	56.16
1, 3	44	12.05	68.22
1, 3, 4	1	0.27	68.49
1, 3, 5	4	1.1	69.59
1, 3, 5, 0	1	0.27	69.86
1, 4	4	1.1	70.96
1, 5	4	1.1	72.05
2	24	6.58	78.63
2, 0	3	0.82	79.45
2, 3	13	3.56	83.01
2, 3, 4	1	0.27	83.29
2, 3, 5	3	0.82	84.11
2, 5	4	1.1	85.21
3	38	10.41	95.62
3, 0	3	0.82	96.44
3, 5	10	2.74	99.18
4	1	0.27	99.45

```
. tab q13_1 ;
      q13_1 |      Freq.     Percent        Cum.
------------+-----------------------------------
          0 |        189       61.97       61.97
          1 |        116       38.03      100.00
------------+-----------------------------------
      Total |        305      100.00

. tab q13_2 ;

      q13_2 |      Freq.     Percent        Cum.
------------+-----------------------------------
          0 |        220       72.13       72.13
          1 |         85       27.87      100.00
------------+-----------------------------------
      Total |        305      100.00

. tab q13_3 ;

      q13_3 |      Freq.     Percent        Cum.
------------+-----------------------------------
          0 |        177       58.03       58.03
          1 |        128       41.97      100.00
------------+-----------------------------------
      Total |        305      100.00
```

**5**	2	0.55	100						
**Total**	365	100			. tab q13_4 ;				
**Note:**					q13_4 | Freq. Percent Cum.				
**1 = Face mask**					------------+-----------------------------------				
**2 = Rubber gloves**					0 | 292 95.74 95.74				
**3 = Disposable gloves**					1 | 13 4.26 100.00				
**4 = Face shield**					------------+-----------------------------------				
**5 = Other PPE**					Total | 305 100.00				
					. tab q13_5 ;				
					q13_5 | Freq. Percent Cum.				
					------------+-----------------------------------				
					0 | 273 89.51 89.51				
					1 | 32 10.49 100.00				
					------------+-----------------------------------				
					Total | 305 100.00				

The Survey Question in English

1. **Since the beginning of the lockdown for the Coronavirus, what was the main source you depended on to get information about disinfection practice to protect the members of your households from the COVID-19?**
2. **What is the main reason for choosing this source to depend on to get information about disinfection practice to protect the members of your households from the COVID-19?**
3. **Since the start of lockdown for the Coronavirus, did you use any of the following resources at least once to get information about the disinfection practice to protect the members of your households from the COVID-19?**
4. **Since the start of lockdown for the Coronavirus, did you clean your household more frequently?**
5. **Since the start of lockdown for the Coronavirus, did you use cleaning products and disinfectants more frequently?**
6. **Since the start of lockdown for the Coronavirus, what are the products did you use for cleaning and disinfecting your household?**
7. **If you used bleach for disinfection during the lockdown, how many times did you use it per day?**
8. **If you used bleach for disinfection during the lockdown, did you mix it with any other products?**
9. **If you used bleach for disinfection during the lockdown, what products did you use to mix with bleach (other than water)?**
10. **How did you use the cleaning products and disinfectants in your household during the lockdown? You can choose all what applies for you?**
11. **Did you use any personal protective equipment for these chemical products while disinfecting your household during the lockdown for Corona virus?**
12. **Please select which PPE did you use while disinfecting your household during the lockdown for Corona virus?**
13. **If you used bleach for disinfection during the lockdown, did you or any other family member in your household experienced any adverse effects to bleach?**
14. **Please mention the number of persons experienced adverse effects to bleach during the lockdown for the Coronavirus.**
15. **Please mention the age of person(s) experienced adverse effects to bleach during the lockdown for the Coronavirus.**
16. **If you used bleach for disinfection during the lockdown, and you or any other family member in your household experienced any adverse effects to bleach, what are these adverse effects?**
17. **If you used bleach for disinfection during the lockdown, and you or any other family member in your household experienced adverse effects to bleach in the eyes, what are these adverse effects?**
18. **When you or any other family member in your household experienced adverse effects to bleach in the eyes, how did you seek help? Please select the first option did you use to ask for help.**
19. **If you used bleach for disinfection during the lockdown, and you or any other family member in your household experienced adverse effects to bleach in the skin, what are these adverse effects?**
20. **When you or any other family member in your household experienced adverse effects to bleach in the skin, how did you seek help? Please select the first option did you use to ask for help.**
21. **If you used bleach for disinfection during the lockdown, and you or any other family member in your household experienced adverse effects to bleach in the respiratory system, what are these adverse effects?**

22. **When you or any other family member in your household experienced adverse effects to bleach in the respiratory system, how did you seek help? Please select the first option did you use to ask for help.**
23. **If you used bleach for disinfection during the lockdown, and you or any other family member in your household experienced adverse effects to bleach in the digestive system, what are these adverse effects?**
24. **When you or any other family member in your household experienced adverse effects to bleach in the digestive system, how did you seek help? Please select the first option did you use to ask for help.**
25. **If you used bleach for disinfection during the lockdown, and you or any other family member in your household experienced any adverse effects to bleach, did you need to go to a hospital or a clinic?**
26. **Did you feel safe while visiting a hospital or a clinic to seek help due to the experience of you or any other family member of adverse effects as a result of using bleach for disinfection during the lockdown?**
27. **Did you use PPE while going or visiting a hospital or a clinic to seek help due to the experience of you or any other family member of adverse effects as a result of using bleach for disinfection during the lockdown?**
28. **Please select the PPE you used while going or visiting a hospital or a clinic to seek help due to the experience of you or any other family member of adverse effects as a result of using bleach for disinfection during the lockdown?**
29. **When you go to the hospital or the clinic to ask for help due to the experience of adverse effects as a result of using bleach for disinfection during the lockdown, did you feel that you received the proper treatment?**
30. **After receiving you or other family members the proper treatment in the hospital or the clinic, did the adverse effects disappear?**
31. **After receiving you or other family members the proper treatment, was you admitted to the hospital?**
32. **Did you need or any other family member to communicate with a poison center to solve your problem?**
33. **The age group for the person who filled the questionnaire?**
34. **The gender of the person who filled the questionnaire?**
35. **The city where the person who filled the questionnaire lives?**
36. **The country where the person who filled the questionnaire lives?**
37. **The highest degree the person who filled the questionnaire earned?**
38. **The occupation of the person who filled the questionnaire.**
39. **The number of family members of the household of the person who filled the questionnaire.**
40. **The number of children (18 years or less) of the household of the person who filled the questionnaire.**
41. **The number elderlies (60 years or more) of the household of the person who filled the questionnaire.**
